# Stepwise Adsorption of Alkoxy‐Pyrene Derivatives onto a Lamellar, Non‐Porous Naphthalenediimide‐Template on HOPG

**DOI:** 10.1002/chem.202004008

**Published:** 2020-10-14

**Authors:** G. Henrieke Heideman, José Augusto Berrocal, Meike Stöhr, E. W. Meijer, Ben L. Feringa

**Affiliations:** ^1^ Stratingh Institute for Chemistry University of Groningen Nijenborgh 4 9747 AG Groningen The Netherlands; ^2^ Institute for Complex Molecular Systems and Laboratory of Macromolecular and Organic Chemistry Eindhoven University of Technology 5600 MB Eindhoven The Netherlands; ^3^ Zernike Institute for Advanced Materials University of Groningen, 9747 AG Groningen, The Netherlands

**Keywords:** adsorption, internal double bonds, long chain-naphthalenediimides, multicomponent self-assembled monolayers, non-porous templates

## Abstract

The development of new strategies for the preparation of multicomponent supramolecular assemblies is a major challenge on the road to complex functional molecular systems. Here we present the use of a non‐porous self‐assembled monolayer from **uC_33_‐NDI‐uC_33_**, a naphthalenediimide symmetrically functionalized with unsaturated 33 carbon‐atom‐chains, to prepare bicomponent supramolecular surface systems with a series of alkoxy‐pyrene (**PyrOR**) derivatives at the liquid/HOPG interface. While previous attempts at directly depositing many of these **PyrOR** units at the liquid/HOPG interface failed, the multicomponent approach through the **uC_33_‐NDI‐uC_33_** template enabled control over molecular interactions and facilitated adsorption. The **PyrOR** deposition restructured the initial **uC_33_‐NDI‐uC_33_** monolayer, causing an expansion in two dimensions to accommodate the guests. As far as we know, this represents the first example of a non‐porous or non‐metal complex‐bearing monolayer that allows the stepwise formation of multicomponent supramolecular architectures on surfaces.

## Graphical abstract

**Supramolecular surface systems**: A non‐porous monolayer of a long‐carbon chain naphthalenediimide serves as template for the stepwise adsorption of a series of alkoxy‐pyrene derivatives at the liquid/HOPG interface. Depositing the guests onto the template causes a severe reorganization of the initial monolayer, as visualized by scanning tunneling microscopy.

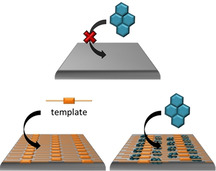

The creation of hierarchical materials and devices by bottom‐up molecular self‐assembly requires the construction of multicomponent supramolecular systems and precise organization at interfaces.[^1^, ^2^, ^3^] Besides novel assembly approaches and tuning of non‐covalent interactions between distinct molecular components, control over hierarchical organization along length scales is among the key topics. Both the design and characterization of multicomponent supramolecular systems still present considerable challenges.[^4^, ^5^] Organic–inorganic hybrid systems like a self‐healable supramolecular polymer,^[6]^ nanoparticle assemblies,^[7]^ or a dual‐mode artificial muscle^[8]^ are illustrative examples for the non‐covalent multicomponent synthesis approach.[^9^, ^10^, ^11^, ^12^, ^13^] Recent developments in supramolecular block copolymers^[14]^ have shown that combining (chiro)optical measurements, fluorescence imaging and computational modeling can furnish insights into these complex multicomponent systems.^[15]^ However, such a deep level of understanding and predictive value in complex systems design probably remains confined to a few specific examples. Confined systems assembled at surfaces bring another level of complexity.[^16^, ^17^, ^18^]

Surface‐supported supramolecular assemblies at the liquid/solid interface are typically studied by scanning tunneling microscopy (STM), which allows to image (multicomponent) self‐assembled monolayers at *quasi*‐molecular resolution.[^1^, ^19^, ^20^, ^21^, ^22^, ^23^, ^24^, ^25^] Although the necessity to induce surface adhesion through chemical design may limit the options to explore, surface‐supported multicomponent supramolecular systems were realized resorting to a limited number of strategies. In particular, porous self‐assembled monolayers have received attention because of their preorganization.[^26^, ^27^, ^28^, ^29^, ^30^, ^31^, ^32^, ^33^, ^34^, ^35^, ^36^, ^37^, ^38^, ^39^] One of the most attractive features of this approach is its modularity, which allows control over pore size and, hence, molecular dimensions of the trapped guests.[^39^, ^40^, ^41^, ^42^, ^43^, ^44^, ^45^] Other reports have provided alternative strategies based on host‐guest interactions with metal complexes.[^46^, ^47^, ^48^] Seeking for alternative strategies/molecular components to allow the stepwise deposition of guest species is key to further expand the fabrication of functional nanostructures and molecular defined surface systems.

Here we report on the templated deposition of pyrene derivatives (**PyrOR**), **PyrOMe**, **PyrSMe**, **PyrOEt**, **PyrOPr** and **PyrOBu**, on the non‐porous self‐assembled monolayer of the long‐carbon chain naphthalenediimide (NDI) **uC_33_‐NDI‐uC_33_** ^[49]^ at the 1‐phenyloctane/highly oriented pyrolytic graphite (1‐PO/HOPG) interface. The choice of the term “non‐porous” refers to the lack of preorganization of the initial **uC_33_‐NDI‐uC_33_** template. In other words, pristine **uC_33_‐NDI‐uC_33_** monolayers do not feature two‐dimensional cavities—areas of uncovered underlying HOPG substrate physically confined by **uC_33_‐NDI‐uC_33_**—onto which the **PyrOR** guests can be physisorbed.

All the chemical structures investigated are shown in Figure 1, whereas their syntheses and characterization are presented in the Supporting Information (Supporting Information, pages S7–S14). Previous investigation highlighted the possibility to deposit pyrene moieties at the liquid/HOPG interface, provided the simultaneous presence of other extended aromatic moieties in the chemical design.[^35^, ^50^] Congruously, all our attempts at the “untemplated” deposition of **PyrOMe**, **PyrSMe**, **PyrOEt** and **PyrOPr** failed. The only exception was **PyrOBu**, which forms self‐assembled monolayers at the 1‐PO/HOPG interface (Supporting Information, Figure S4). Hence, we highlight the use of our **uC_33_‐NDI‐uC_33_** template as a tool to adsorb and organize with nanometer precision alkoxy‐pyrene derivatives on HOPG.

**Figure 1:**
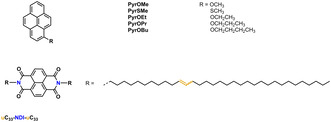
Chemical structures of **PyrOMe**, **PyrSMe**, **PyrOEt**, **PyrOPr**, **PyrOBu**, and **uC_33_‐NDI‐uC_33_**.

We recently showed that long carbon‐chain NDIs (**C_n_‐NDI‐C_n_**) featuring internal double bonds along the carbon‐chain self‐assemble at the 1‐PO/HOPG interface into lamellar monolayers. They comprise of alternating areas of NDI cores and carbon tails, which appear as bright protrusions and darker regions, respectively, in the STM images.^[49]^ The internal double bonds along the carbon chains could also be imaged in the darker areas as additional bright protrusions symmetrically placed with respect to the aromatic cores.^[49]^ The simultaneous presence of *EE*‐, *EZ*‐and *ZZ*‐configured isomers of **uC_33_‐NDI‐uC_33_** did not impede the formation of long‐range ordered lamellar domains, also due to the preferred physisorption for the less abundant *EE*‐configured molecules.^[49]^ Preparation of **uC_33_‐NDI‐uC_33_** monolayers confirmed our previous results,^[49]^ as shown by the STM image and unit cell lattice parameters shown in Figure 2. Right after having obtained the monolayer, we rinsed the modified HOPG with *n*‐octanoic acid (OA) to remove the excess of **uC_33_‐NDI‐uC_33_** that did not adhere to the substrate and checked the preservation of the monolayer after the rinsing step by STM (Supporting Information, Figure S1).

**Figure 2:**
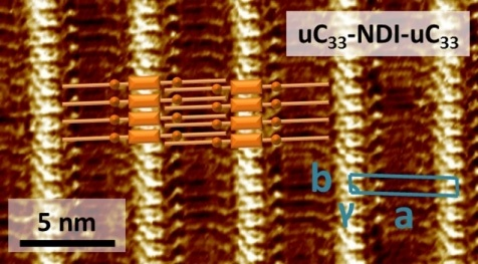
STM image of **uC_33_‐NDI‐uC_33_** at the 1‐PO/HOPG interface, with a schematic representation of the packing in which the orange rectangular shapes represent the NDI core, the orange lines the carbon chains and the orange dots the internal double bonds. The unit cell and the lattice parameters^[49]^ **a**, **b** and **γ** are depicted in blue.

Next, the rinsed **uC_33_‐NDI‐uC_33_** monolayer was treated with a **PyrOMe** solution in OA. STM imaging of the newly treated surface revealed the presence of new protrusions placed in a zipper‐like fashion alongside the NDI cores of the original lamellar morphology (Figure 3 a). These new protrusions were more extended and brighter than the internal double bonds of **uC_33_‐NDI‐uC_33_** of Figure 2.

**Figure 3:**
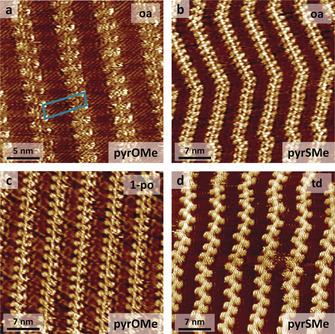
STM images of **uC_33_‐NDI‐uC_33_** monolayers after the deposition of: a) **PyrOMe** (OA/HOPG interface; 25 nm×25 nm, *V*_tip_=1 V, *I*_set_=50 pA); b) **PyrSMe** (OA/HOPG interface; 35 nm×35 nm, *V*_tip_=1 V, *I*_set_=100 pA); c) **PyrOMe** (1‐PO/HOPG interface; 35 nm×35 nm, *V*_tip_=1 V, *I*_se_t=60 pA); d) **PyrSMe** (TD/HOPG interface; 35 nm×35 nm, *V*_*t*ip_=1 V, *I*_set_=100 pA). The schematic unit cell of the **PyrOMe/uC_33_‐NDI‐uC_33_** bicomponent system is displayed as a blue rectangle in Figure 3 a as a guide to the eye.

Intrigued by this preliminary result, we repeated the experiment with **PyrSMe**, the sulfur analogue of **PyrOMe**, to examine the possible consequences on both adsorption and imaging contrast deriving from the substitution of the heteroatom in the (thio)ether linkage. Similar STM images were recorded, with the new bright dots ascribed to **PyrSMe** staying next to the NDI cores in a zipper‐like fashion (Figure 3 b). Replacing OA with 1‐PO or *n*‐tetradecane (TD) consistently afforded the same results with both **PyrOMe** (Figure 3 c) and **PyrSMe** (Figure 3 d), suggesting no influence of the solvent(s) chosen to form the bicomponent system and the robustness of the multicomponent co‐assembly. Time monitoring of the sequential adsorption of **PyrOMe**/**PyrSMe** onto the **uC_33_‐NDI‐uC_33_** template suggested the occurrence of nucleation processes: low coverages by **PyrOMe**/**PyrSMe** were imaged at the initial stages of the process (5 min), with clear surface regions showing only the initial **uC_33_‐NDI‐uC_33_** monolayer (Supporting Information, Figure S2). Prolonged exposure (3–4 hours) of the **uC_33_‐NDI‐uC_33_** template to the **PyrOMe**/**PyrSMe** solutions resulted in quantitative coverage (Figure 3).

Having demonstrated the consistency of our systems over a broad scope of solvents (OA, 1‐PO and TD), we introduced subtle structural modifications in the **PyrOR** molecular design. Extending the length of the alkyl chain by one (**PyrOEt**) or two (**PyrOPr**) carbon atoms resulted in the same outcome observed with **PyrOMe** and **PyrSMe**, namely the zipper‐like deposition of the pyrene derivatives alongside the NDI cores of **uC_33_‐NDI‐uC_33_** (Figure 4 a for **PyrOEt**, Figure 4 b for **PyrOPr**). In stark contrast, **PyrOBu** offered only modest signs of adsorption onto the **uC_33_‐NDI‐uC_33_** template at ca. 10^−2^ m concentrations (Supporting Information, Figure S4). A ten‐fold increase in concentration resulted in the displacement of **uC_33_‐NDI‐uC_33_** and subsequent formation of a new monolayer uniquely formed by **PyrOBu**, instead (Supporting Information, Figure S4). Such observation suggested that the competition between stepwise adsorption onto the **uC_33_‐NDI‐uC_33_** template and alkoxy‐pyrene monolayer formation is controlled by the subtle interplay of different parameters, which certainly include concentration and adsorption energy of the alkoxy‐pyrenes on HOPG. This balance becomes particularly evident in the case of **PyrOBu**, which features a longer alkyl chain and likely enhanced van der Waals interactions, since the other **PyrOR** investigated do not form monolayers at the liquid/HOPG interface. Indeed, the use of more concentrated (saturated) solutions of **PyrOMe**, **PyrSMe**, **PyrOEt** and **PyrOPr** did not affect their adsorption onto the **uC_33_‐NDI‐uC_33_** template.

**Figure 4:**
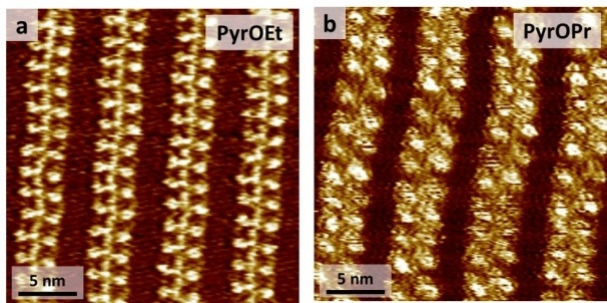
STM images after the sequential deposition onto the **uC_33_‐NDI‐uC_33_** template in OA of: a) **PyrOEt** (25 nm × 25 nm, *V*_tip_=1 V, *I*_set_=10 pA), and b) **PyrOPr** (25 nm×25 nm, *V*_tip_=1.4 V, *I*_set_=15 pA).

In addition, we tested other potential templates from the unsaturated and saturated **C_n_‐NDI‐C_n_**[^49^, ^51^] family to elucidate essential structural parameters (the complete list of **C_n_‐NDI‐C_n_** tested is reported in Figure S5). Surprisingly enough, although these compounds are structurally very similar to **uC_33_‐NDI‐uC_33_**, their assistance towards subsequent adsorption of the **PyrOR** compounds was extremely modest. The case of **C_33_‐NDI‐C_33_** ^[49]^ (Figure S5)_,_ which only differs from **uC_33_‐NDI‐uC_33_** by the absence of the internal double bonds in the chemical structure, is particularly remarkable. Currently, the reason for the high selectivity of the **uC_33_‐NDI‐uC_33_** monolayer is not understood, but it certainly highlights a “unique” character for this particular template. Importantly, all the experimental results on co‐assembly obtained with the **uC_33_‐NDI‐uC_33_** monolayer were highly consistent and reproducible.

Further insights into the system were obtained by analyzing the profile plots of the STM images obtained during the early stages (5 min) of the **PyrSMe** adsorption (Figure 5). The coverage of the **uC_33_‐NDI‐uC_33_** adlayer by **PyrSMe** was not quantitative during the initial stages of the process. Thus, the STM images displayed the simultaneous presence of the **uC_33_‐NDI‐uC_33_** lamellae similar to Figure 2, and additional protrusions positioned in a zipper‐like fashion with respect to the NDI cores as in Figures 3 and 4 (STM image of Figure 5). Profile plots measured over lengths of approximately 42 nm in both surface areas, that is, with and without adsorbed **PyrSMe**, highlighted remarkable differences in the periodical organizations. The underlying **uC_33_‐NDI‐uC_33_** template was highly regular and consistent with an about 5 nm periodical lamellar morphology in accordance with our previous report^[49]^ (orange trace in Figure 5, bottom). The profile plot of the surface area featuring the adsorbed **PyrSMe** showed less regularity, although a clear increase in the distance between the maxima was noticeable (cyan trace in Figure 5, top). The presence of 7 maxima in 42 nm resulted in a 6 nm average distance between parallel arrays in the surface areas with **PyrSMe**. This suggested that the parallel NDI cores increased their distance by approximately 1 nm to host **PyrSMe** and form the bicomponent system.

**Figure 5:**
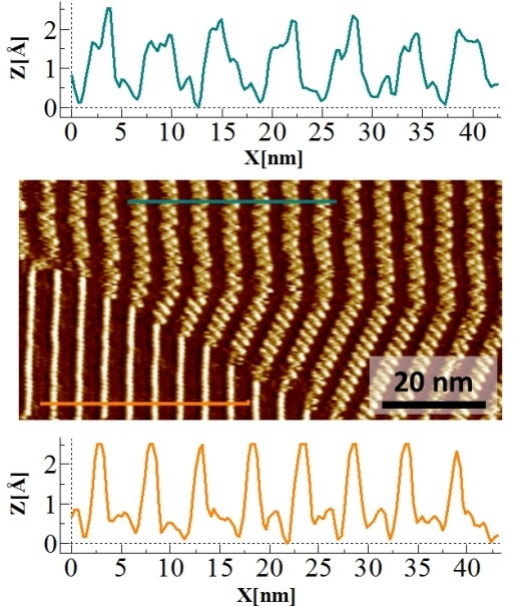
Profile plots measured over approximately 42 nm of the STM image (middle) of **PyrSMe** adsorption within the **uC_33_‐NDI‐uC_33_** template in OA (*V*_tip_=1 V, *I*_set_=50 pA), showing seven maxima (6 nm periodicity) after adsorption of **PyrSMe** (top, cyan) and eight maxima (5 nm periodicity) in the original **uC_33_‐NDI‐uC_33_** monolayer (bottom, orange).

Combining all the information obtained, we formulate a schematic pictorial representation for the **PyrOMe**/**uC_33_‐NDI‐uC_33_** bicomponent system (Figure 6). Although the clear‐cut image in Figure 5 features **PyrSMe**, the qualitative model was built for **PyrOMe** due to the larger statistics available for such **PyrOR**. While the cartoon depicts a regular system, we would like to stress that analysis of the STM images revealed fluctuations in the molecular arrangement. Hence, the illustration in Figure 6 should be taken as a qualitative description rather than an unambiguous unit cell of the **PyrOMe**/**uC_33_‐NDI‐uC_33_** monolayer. Moreover, the orientation of the alkoxy‐pyrene moieties was arbitrarily chosen in our qualitative model due to the lack of precise information obtained from the STM images. A previous model to explain the deposition of pyrenes in porous templates followed the same approach.^[52]^ Statistical analysis on a number of STM images of the **PyrOMe**/**uC_33_‐NDI‐uC_33_** system allowed to estimate an **a_PyrOMe_** value as high as 6.05±0.15 nm (averaged value for the assemblies in 1‐PO and OA), whereas the **a** unit cell parameter for the original **uC_33_‐NDI‐uC_33_** monolayer is 5.27±0.08 nm (**a** is shown in Figure 2).^[49]^ The enhancement of the lateral distance between two rows of NDIs in the bicomponent system (**a_PyrOMe_**) pointed to a lateral expansion of the initial **uC_33_‐NDI‐uC_33_** monolayer to accommodate **PyrOMe**. Since the unsaturated carbon chains of **uC_33_‐NDI‐uC_33_** have a strong preference for interdigitation,^[49]^ we hypothesize that such lateral expansion necessarily pulls the carbon chains apart and creates exposed pockets at well‐defined distances on the underlying HOPG substrate, while partially retaining the interdigitation of the template molecules. The lattice expansion along the **a** vector appears to reach a maximum limit, which explains the decreased adsorption of **PyrOR** upon chain extension. A further expansion along the **b** direction is also necessary to favor the adsorption of **PyrOMe**, modifying the **b** unit cell parameter from 0.94±0.06 nm to an average value 1.84±0.22 for **b_PyrOMe_** (obtained from the statistical analysis of a number of STM images 1‐PO and OA). The two‐dimensional expansion of the **uC_33_‐NDI‐uC_33_** template is in line with the lack of preorganization of the initial monolayer. Indeed, the latter must adapt to the presence of the **PyrOR** species and create the pockets for their adsorption. Additional stabilizing intermolecular interactions between pyrenes and NDI cores cannot be excluded within the bicomponent system. However, a hypothesis on the exact geometry would be speculative due to the unknown orientation of the pyrene derivatives and awaits in depth computational studies. Nevertheless, it should be emphasized that the positioning of alkoxy‐pyrenes alongside the NDI cores, and not in the typical stacked donor‐acceptor configuration occurring in charge‐transfer solution‐phase supramolecular systems,^[53]^ is a unique aspect of our system.

**Figure 6:**
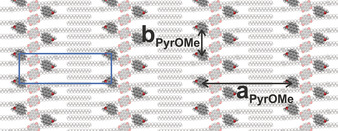
Qualitative representation of the **PyrOMe**/**uC_33_‐NDI‐uC_33_** bicomponent system with related **a_PyrOMe_** and **b_PyrOMe_** distances (6.05±0.15 and 1.84±0.22 nm, respectively). The blue rectangle shown in Figure 3 as guide to the eye is also displayed.

In conclusion, we presented the stepwise adsorption of alkoxy‐pyrene derivatives **PyrOMe**, **PyrSMe**, **PyrOEt**, **PyrOPr** and **PyrOBu** onto a lamellar, non‐porous **uC_33_‐NDI‐uC_33_** template on HOPG. The deposition of these alkoxy‐pyrenes brings to the formation of a bicomponent system that appears dramatically different from the initial monolayer upon STM imaging. New bright protuberances (the alkoxy‐pyrenes) are imaged in a zipper‐like fashion alongside the NDI cores. Moreover, the formation of a bicomponent system significantly alters the organization of the initial **uC_33_‐NDI‐uC_33_** template, causing an expansion in both directions of the original unit cell directions to accommodate the guest in the template. To the best of our knowledge, the **uC_33_‐NDI‐uC_33_** template is the first self‐assembled monolayer that allows for the stepwise construction of on‐surface multicomponent supramolecular architectures without resorting to the preorganization of porous architectures or host‐guest interactions with metal‐complexes. This offers a unique approach to establish future directions of supramolecular surface chemistry through stepwise multicomponent assembly. Current efforts of our joined research program focus on the use of the **uC_33_‐NDI‐uC_33_** template as a molecular track for the controlled unidirectional translation of light‐driven molecular motors on surfaces.

## Conflict of interest

The authors declare no conflict of interest.

## Supporting information

As a service to our authors and readers, this journal provides supporting information supplied by the authors. Such materials are peer reviewed and may be re‐organized for online delivery, but are not copy‐edited or typeset. Technical support issues arising from supporting information (other than missing files) should be addressed to the authors.

Supplementary
