# Supplementary material for: Stepwise Adsorption of Alkoxy‐Pyrene Derivatives onto a Lamellar, Non‐Porous Naphthalenediimide‐Template on HOPG
Source: Chemistry. 2020 Oct 14;27(1):207–11. doi: 10.1002/chem.202004008 (PMC7821129; doi:10.1002/chem.202004008)
Supplement: Supplementary file 1 — Supplementary [file CHEM-27-207-s001.pdf]

Chemistry–A European Journal

Supporting Information

Stepwise Adsorption of Alkoxy-Pyrene Derivatives onto a Lamellar, Non-Porous Naphthalenediimide-Template on HOPG

G. Henrieke Heideman+,[a] José Augusto Berrocal+,[a, b] Meike Stöhr,[c] E. W. Meijer,*[b] and Ben L. Feringa*[a]

Table of Contents

Experimental (STM) S3
Additional data S3
Figure S1 .... S3
Figure S2 .... S4
Figure S3 .... S4
Selectivity of the template S5
Figure S4 .... S5
Investigation different templates S6
Figure S5 .... S6
Figure S6 .... S6
Experimental (synthesis) S7
References S15

Experimental (STM)

All experiments were performed at room temperature (21-25 °C) using a Molecular Imaging STM operating in constant-current mode at the solvent/HOPG interface. STM tips were prepared by mechanical cutting of Pt/Ir wire (90/10, diameter 0.25 mm, Goodfellow). The solution of the template molecules was prepared by dissolving uC_{33}-NDI-uC_{33} (0.4 mg/ml) in 1-phenyloctane (>98.0%, purchased by TCI). The NDI solution was heated to 100 °C and subsequently drop casted onto a freshly cleaved HOPG surface (ZYB grade, Bruker AFM probes). Next, the uC_{33}-NDI-uC_{33} layer was vigorously rinsed with the solvent used in the pyrene adsorption experiments (1-phenyloctane, *n*-octanoic acid or *n*-tetradecane) and imaged with the STM before the deposition of the pyrene-derivatives. In order to perform the STM measurements it was required that the adsorbates were as pure as possible. PyrOMe (1.9×10^{-1} mbar at 130°C) and PyrOPr (4.0×10^{-2} mbar at 155°C) were purified by sublimation prior to the STM experiments. PyrOEt and PyrSMe were recrystallized from MeOH prior to the STM experiments. PyrOBu was further purified by another flash column chromatography (pentane/DCM(0-10%) in order to obtain a white solid. The purified alkoxy-pyrene derivatives were dissolved in 1-phenyloctane (>98.0%, purchased by TCI), *n*-octanoic acid (>98.0%, purchased by TCI) or *n*-tetradecane (>99.5%, purchased by TCI) and drop casted on top of uC_{33}-NDI-uC_{33} at room temperature. All STM images were analyzed and processed using WSxM 5.0.[1] All bias values are given with respect to a grounded tip.

Additional data

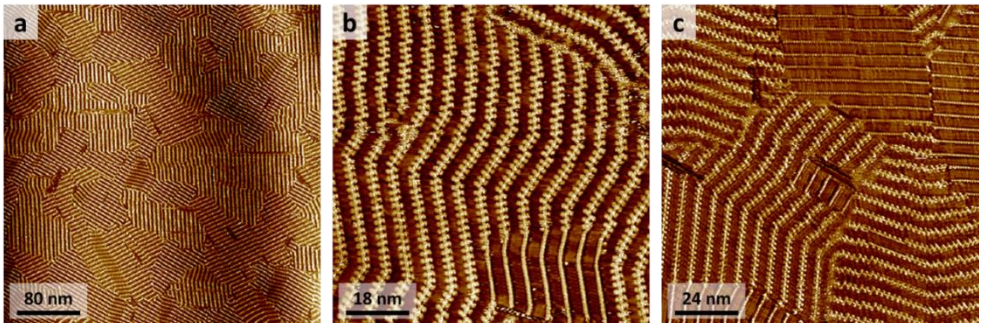

Figure S1. STM images of **PyrOMe** adsorption experiments in 1-phenyloctane (1-PO): a) **uC_{33}-NDI-uC_{33}** after the rinsing step (400 nm × 400 nm, V_{tip} = 1 V, I_{set} = 100 pA). b) Adsorbed **PyrOMe** (saturated solution 10× diluted) on the **uC_{33}-NDI-uC_{33}** layer at the 1-PO/HOPG interface. Showing a small island of bare **uC_{33}-NDI-uC_{33}** in the bottom right corner (90 nm × 90 nm, V_{tip} = 1 V, I_{set} = 50 pA). c) Adsorbed **PyrOMe** (saturated solution 10× diluted) on the **uC_{33}-NDI-uC_{33}** layer at the 1-PO/HOPG interface, showing islands of bare **uC_{33}-NDI-uC_{33}** in the top right corner (120 nm × 120 nm, V_{tip} = 1 V, I_{set} = 60 pA). Adsorption of **PyrOMe** took place within 7 minutes after deposition.

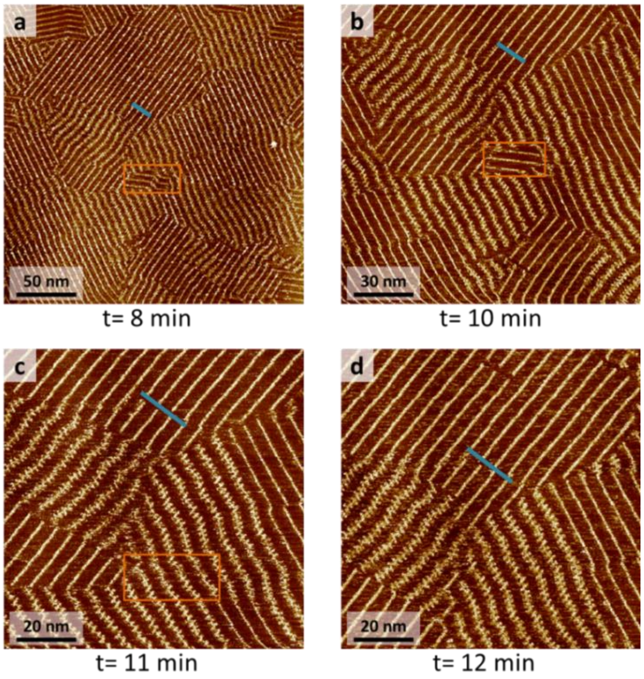

Figure S2. **PyrSMe** (8×10^{-3} M in *n*-tetradecane) adsorption on **uC$_{33}$-NDI-uC$_{33}$** template followed over time. The time underneath the images corresponds to the time lapsed after the **PyrSMe** deposition. The turquoise marker is placed on all the images on the same spot of the surface. More **PyrSMe** molecules were adsorbed over time in the area indicated by the orange box. Imaging parameters a) 250 nm × 250 nm, V_{tip} = 1 V, I_{set} = 100 pA; b) 150 nm × 150 nm, V_{tip} = 1 V, I_{set} = 100 pA; c) 100 nm × 100 nm, V_{tip} = 1 V, I_{set} = 100 pA and; d) 100 nm × 100 nm, V_{tip} = 1 V, I_{set} = 50 pA.

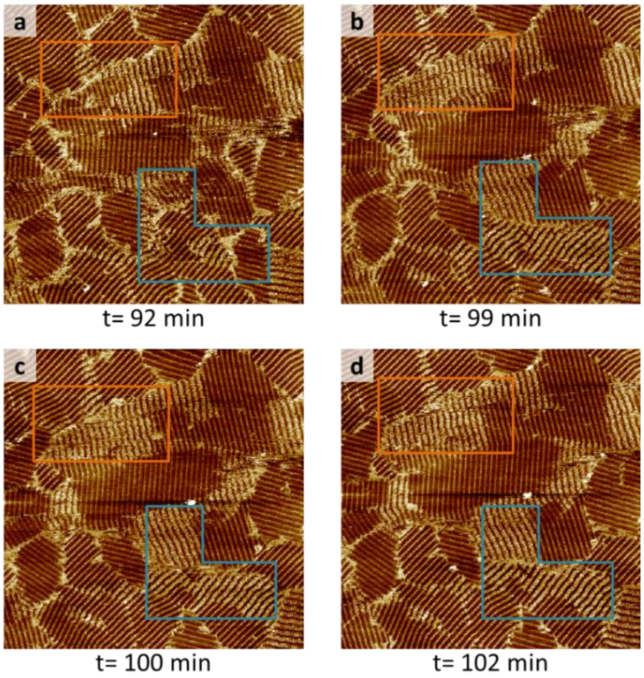

Figure S3. PyrOPr adsorption on **uC$_{33}$-NDI-uC$_{33}$** template followed over time (solution: saturated solution in *n*-octanoic acid 2 to 3 times diluted). The time underneath the images corresponds to the time lapsed after the deposition of the **PyrOPr** deposition. a-d) imaging parameters: 300 nm × 300 nm, V_{tip} = 0.8 V, I_{set} = 25 pA.

Selectivity of the template

The adsorption of **PyrOMe**, **PyrSMe** and **PyrOEt** on the **uC$_{33}$-NDI-uC$_{33}$** template was highly favored. However, the adsorption of **PyrOPr** was more difficult, leading to a limited amount of surface coverage by **PyrOPr**. Moreover, the **PyrOPr**/**uC$_{33}$-NDI-uC$_{33}$** bicomponent system was more difficult to resolve. Finally, **PyrOBu** was also studied, as described in the main text.

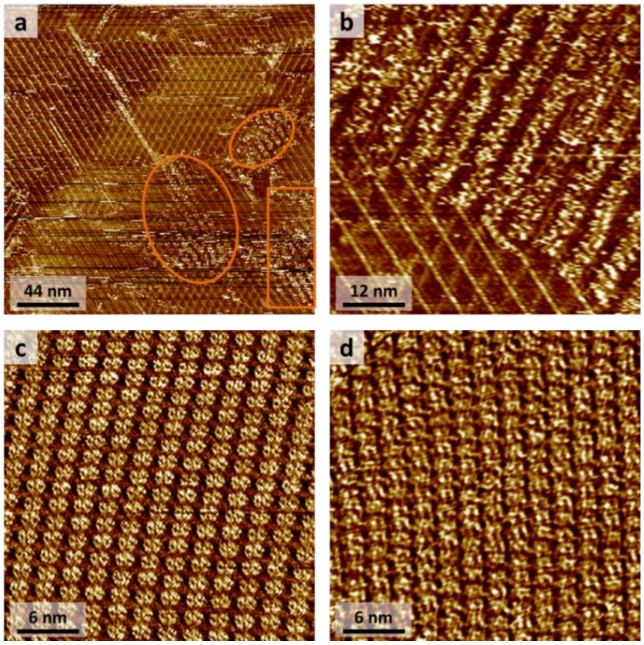

Figure S4. STM images after the adsorption of **PyrOBu**. a) STM image after deposition of **PyrOBu** (6.5×10^{-2}M in *n*-octanoic acid) on the **uC$_{33}$-NDI-uC$_{33}$** template, with only small island of trapped **PyrOBu** molecules observed (marked with orange circles). Imaging parameters: 220 nm × 220 nm, V_{tip} = 1.4 V, I_{set} = 25 pA. b) STM image after deposition of **PyrOBu** (6.5×10^{-2}M in *n*-octanoic acid) on the **uC$_{33}$-NDI-uC$_{33}$** template, with lowered resolution on the surface areas with adsorbed **PyrOBu** (60 nm × 60 nm, V_{tip} = 1.4 V, I_{set} = 25 pA). c) STM image after deposition of **PyrOBu** (1.6×10^{-1}M in *n*-octanoic acid) on the **uC$_{33}$-NDI-uC$_{33}$** template, showing highly resolved **PyrOBu** molecules, suggesting replacement of **uC$_{33}$-NDI-uC$_{33}$** by **PyrOBu** (30 nm × 30 nm, V_{tip} = 1.3 V, I_{set} = 15 pA). d) STM image after the deposition of **PyrOBu** (1.6×10^{-1}M in *n*-octanoic acid) on bare HOPG, showing the same qualitative pattern observed in image c (30 nm × 30 nm, V_{tip} = 1.0 V, I_{set} = 25 pA).

Investigation of different potential templates

All **uC$_n$-NDI-uC$_n$** adlayers were prepared using reported procedures.[2] After rinsing, either **PyrOMe** in *n*-octanoic acid or **PyrSMe** in *n*-tetradecane were added to test the different templates. Only traces of adsorbed pyrenes could be observed on **u$_2$C$_{39}$-NDI-u$_2$C$_{39}$**[3] and **u$_3$C$_{50}$-NDI-u$_3$C$_{50}$**[3] adlayers, while no pyrene molecules were observed on the **uC$_{28}$-NDI-uC$_{28}$**[2] and on the **C$_{33}$-NDI-C$_{33}$**[2] adlayer.

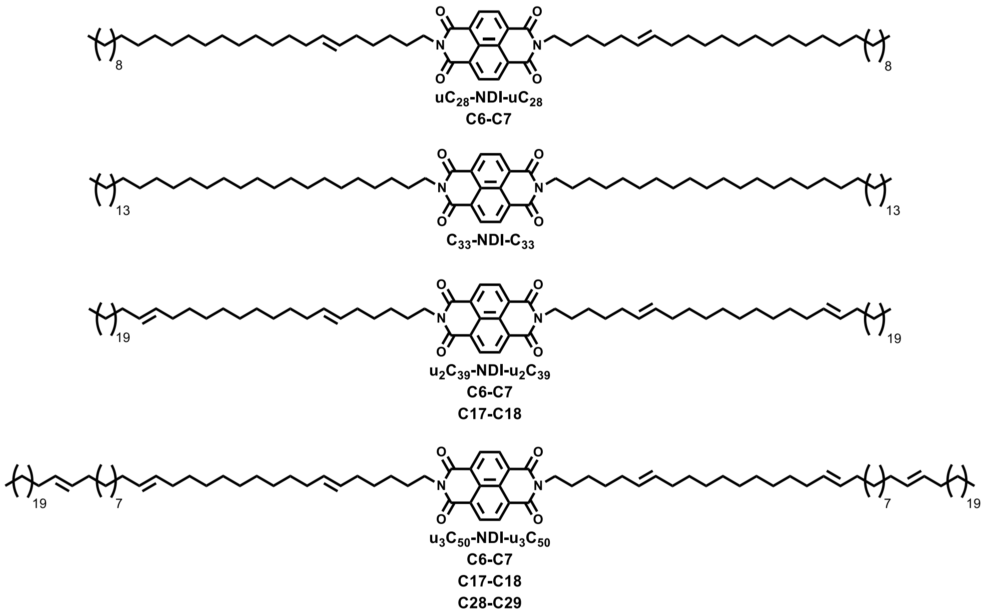

Figure S5. Chemical structures of different alkylated NDI molecules. The subscripts (C(x)-C(y)) refers to the positions of the double bond in the carbon chains.

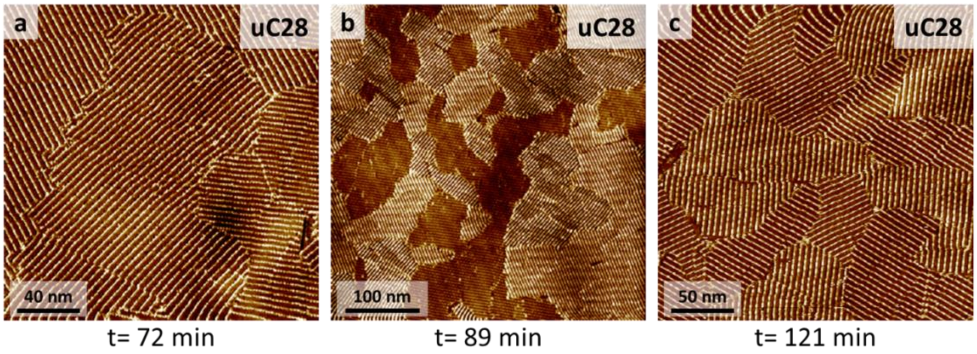

Figure S6. Adsorption experiments with **PyrSMe** (8×10^{-3} M in *n*-tetradecane) on the **uC$_{28}$-NDI-uC$_{28}$** adlayer. The time underneath the images corresponds to the time lapsed after the deposition of **PyrSMe**. No significant changes of the adlayer were detected overtime. a) Imaging parameters: 200 nm × 200 nm, V_{tip} = 1 V, I_{set} = 100 pA. b) Imaging parameters: 500 nm × 500 nm, V_{tip} = 1 V, I_{set} = 100 pA. c) Imaging parameters: 250 nm × 250 nm, V_{tip} = 1 V, I_{set} = 100 pA.

Experimental (synthesis)

1-methoxypyrene (PyrOMe): 1-hydroxypyrene (501 mg, 2.3 mmol) was added to a mixture of Na_2CO_3 (900 mg, 8.4 mmol) and MeI (540 mg, 3.8 mmol) in DMF (15 mL) under nitrogen atmosphere. The reaction mixture was heated to 60°C and stirred overnight. The mixture was diluted in ether (100 mL) after cooling to rt and washed with brine (2× 150 ml). The aqueous phases were combined and extracted with ether (150 mL). The organic layer was subsequently washed with brine and dried over $MgSO_4$ prior to the evaporation of the solvent under reduced pressure. The residue was purified by flash column chromatography (pentane/DCM(0-20%) to yield 1-methoxypyrene (475 mg, 2.0 mmol, 89% yield) as a white solid. mp: 88.4°C. ^{1}H NMR (400 MHz, $CDCl_3$): δ (ppm) 8.46 (d, J = 9.2 Hz, 1H), 8.13 – 8.04 (m, 4H), 7.98 – 7.94 (m, 2H), 7.89 (d, J = 8.9 Hz, 1H), 7.56 (d, J = 8.4 Hz, 1H), 4.18 (s, 3H).^{13}C NMR (101 MHz, $CDCl_3$): δ (ppm) 153.81, 131.90, 131.84, 127.39, 126.53, 126.24, 125.95, 125.61, 125.40, 125.12, 125.09, 124.38, 124.29, 121.27, 120.38, 108.22, 56.29. HRMS (ESI+, m/z): Calcd for $C_{17}H_{13}O$ [M+H$^+$]: 233.0966, found: 233.0959. These results matches with previously reported data on 1-methoxypyrene.[4–7]

1-ethoxypyrene (PyrOEt): 1-hydroxypyrene (500 mg, 2.3 mmol) was added to a mixture of Na_2CO_3 (600 mg, 5.6 mmol) and ethyl iodide (430 mg, 2.8 mmol) in DMF (15 ml) under nitrogen atmosphere. The reaction mixture was stirred at 60°C overnight. To push the reaction to full conversion, excess Na_2CO_3 (200 mg, 1.9 mmol) and ethyl iodide (978 mg, 6.3 mmol) was added and the reaction mixture was allowed to cool down to rt 4 hours later. The mixture was dissolved in EtOAc (50 ml) and washed with H_2O (50 mL). The organic layer was subsequently washed with H_2O (50 ml) and brine (50 ml) and dried over $MgSO_4$. The volatiles were removed *in vacuo* and the residue was recrystallized from MeOH* prior to purification by flash column chromatography (pentane/DCM(0-10%) yielding in an off white solid. The solid was recrystallized twice in MeOH and EtOH to yield a white solid (271 mg, 1.1 mmol, 49 %). mp: 71.0 °C (lit. 72-73 °C).[8] ^{1}H NMR (400 MHz, $CDCl_3$): δ (ppm) 8.50 (d, J = 9.1 Hz, 1H), 8.13 – 8.04 (m, 4H), 7.98– 7.94 (m, 2H), 7.89 (d, J = 8.9 Hz, 1H), 7.54 (d, J = 8.4 Hz, 1H), 4.40 (q, J = 6.9 Hz, 2H), 1.64 (t, J = 7.0 Hz, 3H). ^{13}C NMR (101 MHz, $CDCl_3$): δ (ppm) 153.25, 131.90, 131.87, 127.40, 126.40, 126.20, 126.00, 125.63, 125.31, 125.13, 125.05, 124.32, 124.24, 121.47, 120.59, 109.34, 77.48, 77.16, 76.84, 64.71, 15.23. HRMS (ESI+, m/z): Calcd for $C_{18}H_{15}O$ [M+H$^+$]: 247.1123, found: 247.1128.

*Compound was pure by ^{1}H NMR after the first recrystallization but was further purified for the use in the STM experiments.

1-propoxypyrene (PyrOPr): 1-hydroxypyrene (507 mg, 2.3 mmol) was added to a mixture of Na_2CO_3 (728 mg, 6.8 mmol) and propyl iodide (590 mg, 3.4 mmol) in DMF (15 mL) under nitrogen atmosphere. The reaction mixture was stirred at 60°C overnight. To push the reaction to full conversion, excess of Na_2CO_3 (150 mg, 1.4 mmol) and propyl iodide (100 mg, 0.6 mmol) was added and the reaction mixture was allowed to cool down to rt 4 hours later. The mixture was diluted in ether (100 mL) and washed with half saturated brine (2× 150 ml). The combined aqueous phases were extracted with ether and the organic layer was washed one more time with brine. The organic layer was dried over $MgSO_4$ before the volatiles were removed *in vacuo*. The residue was purified by flash column chromatography (pentane/DCM(0-20%) to yield 1-propoxypyrene (469 mg, 1.8 mmol, 78% yield) as a white solid. mp: 81.2 °C (lit. 78-79 °C).[8] ^{1}H NMR (400 MHz, $CDCl_3$): δ (ppm)8.51 (d, J = 9.2 Hz, 1H), 8.13 – 8.04 (m, 4H), 7.98 – 7.95 (m, 2H), 7.89 (d, J = 9.0 Hz, 1H), 7.53 (d, J = 8.4 Hz, 1H), 4.29 (t, J = 6.4 Hz, 2H), 2.05 (m, 2H), 1.22 (t, J = 7.4 Hz, 3H). ^{13}C NMR (101 MHz, $CDCl_3$): δ (ppm) 153.38, 131.90, 131.87, 127.40, 126.39, 126.18, 126.00, 125.63, 125.25, 125.14, 125.01, 124.30, 124.21, 121.44, 120.58, 109.30, 70.61, 23.03, 10.95. HRMS (ESI+, m/z): Calcd for $C_{19}H_{17}O$ [M+H$^+$]: 261.1279, found: 261.1274. These results matches with previously reported data on 1-propoxypyrene.[7]

1-butoxypyrene (PyrOBu): 1-hydroxypyrene (500 mg, 2.3 mmol) was added to a mixture of Na_2CO_3 (610 mg, 5.7 mmol) and 1-bromobutane (381 mg, 2.8 mmol) in DMF (75 ml) under nitrogen atmosphere. The reaction mixture was stirred at 60°C for 48 hours. The mixture was diluted in ether and washed with H_2O. The organic layer was washed with brine (2×) and dried over $MgSO_4$. The volatiles were removed *in vacuo* and the residue was purified by flash column chromatography (pentane/EtOAc (0-10%) to yield 1-butoxypyrene (440 mg, 1.6 mmol, 70% yield) as an off white solid. mp: 87.7 °C (lit. 87 °C).[8] ^{1}H NMR (400 MHz, $CDCl_3$): δ (ppm) 8.48 (d, J = 9.2 Hz, 1H), 8.12-8.08 (m, 3H), 8.03 (d, J = 9.2 Hz, 1H), 7.97 – 7.93 (m, 2H), 7.88 (d, J = 8.9 Hz, 1H), 7.55 (d, J = 8.4 Hz, 1H), 4.34 (t, J = 6.4 Hz, 2H), 2.05 – 1.97 (m, 2H), 1.73 – 1.63 (m, 2H), 1.08 (t, J = 7.4 Hz, 3H). ^{13}C NMR (101 MHz, $CDCl_3$): δ (ppm) 153.43, 131.92, 131.88, 127.41, 126.40, 126.19, 126.01, 125.64, 125.26, 125.15, 125.02, 124.31, 124.22, 121.47, 120.60, 109.30, 68.84, 31.75, 19.67, 14.12. HRMS (ESI+, m/z): Calcd for $C_{20}H_{19}O$ [M+H$^+$]: 275.1436, found: 275.1430.

methyl 1-pyrenyl sulfide (PyrSMe) A solution of 1-bromopyrene (500 mg, 2.3 mmol) in dry THF (25 mL) was cooled down to -78°C. n-Buli (1 ml, 2.5 mmol, 2.5 M in hexane) was added to the solution. The solution was stirred for 30 min, then dimethyldisulfide (237 mg, 2.5 mmol) was added and the solution was stirred for other 15 min. The reaction mixture was allowed to warm up to rt, then it was quenched with 2 mL MeOH. The volatiles were removed *in vacuo* and the residue was dissolved in DCM (50 mL) and washed with brine (2× 50 mL). The organic layer was dried over $MgSO_4$ and the volatiles were removed *in* vacuo. The residue was purified using flash column chromatography (pentane/DCM (0-20%) to yield methyl 1-pyrenyl sulfide (315 mg, 1.3 mmol, 55% yield) as an off white solid. mp: 73.7 °C. ^{1}H NMR (400 MHz, $CDCl_3$): δ (ppm) 8.56 (d, J = 9.2 Hz, 1H), 8.19 – 8.10 (m, 4H), 8.03 – 7.98 (m, 4H), 2.71 (s, 3H). ^{13}C NMR (101 MHz, $CDCl_3$): δ (ppm) 133.16, 131.56, 131.14, 129.47, 129.40, 127.70, 127.37, 127.01, 126.22, 125.37, 125.18, 125.07, 124.64, 123.83, 17.36. HRMS (ESI+, m/z): Calcd for $C_{17}H_{12}S$ [M]$^+$: 248.0660, found: 248.0651. Synthesis was adapted from reference [10].

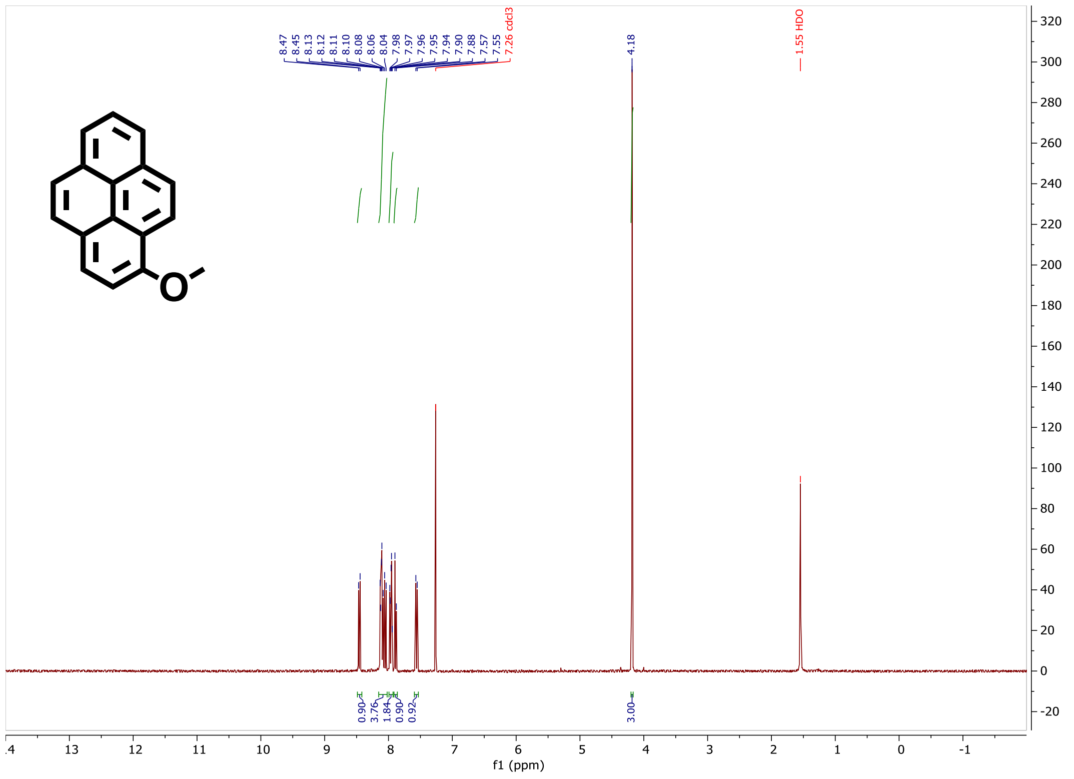

Figure S7. ^{1}H-NMR spectrum of **PyrOMe**.

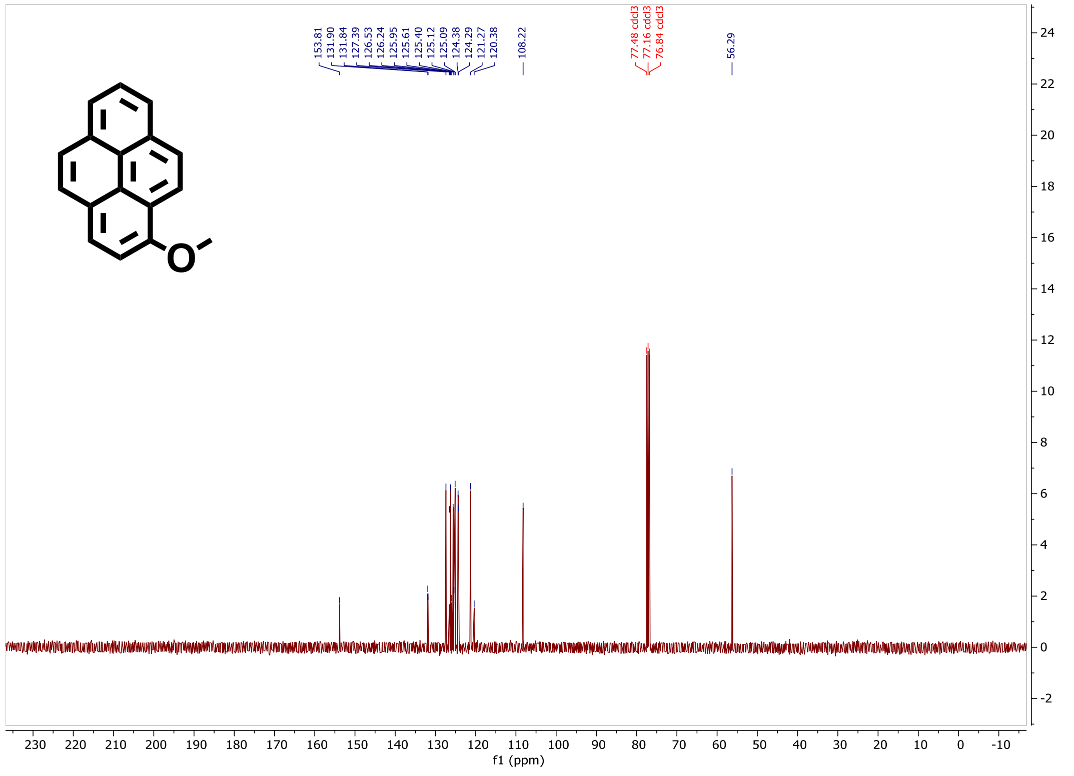

Figure S8. ^{13}C-NMR spectrum of **PyrOMe**.

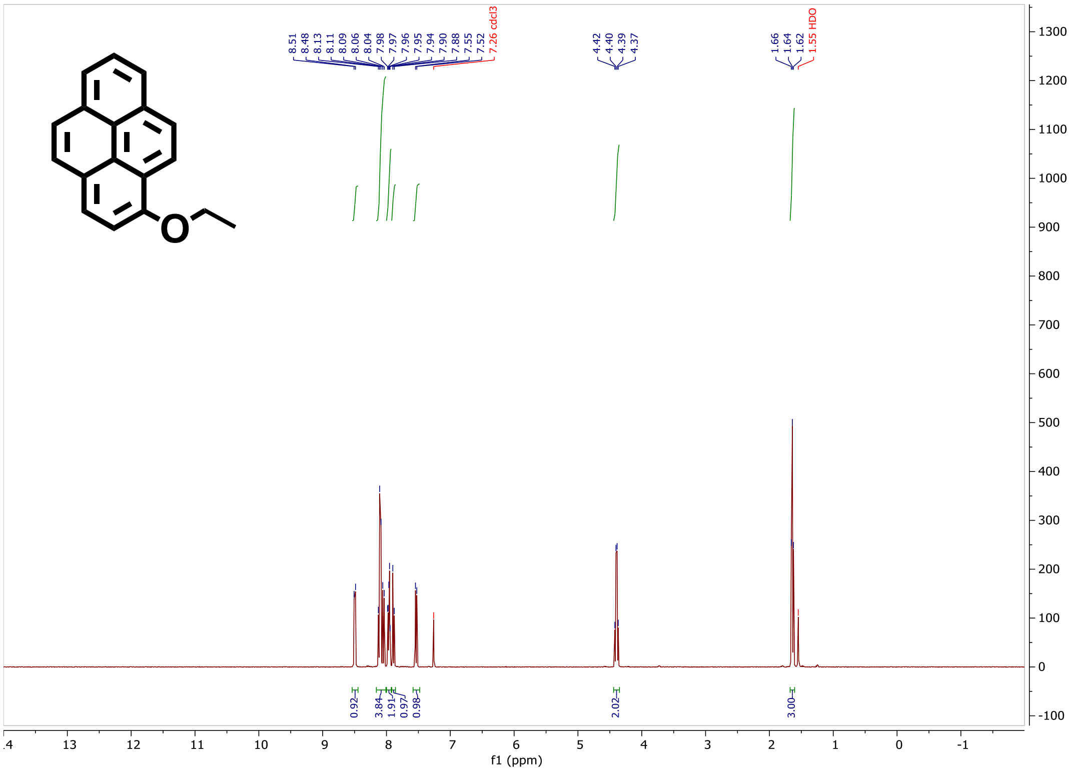

Figure S9. ^{1}H-NMR spectrum of **PyrOEt**.

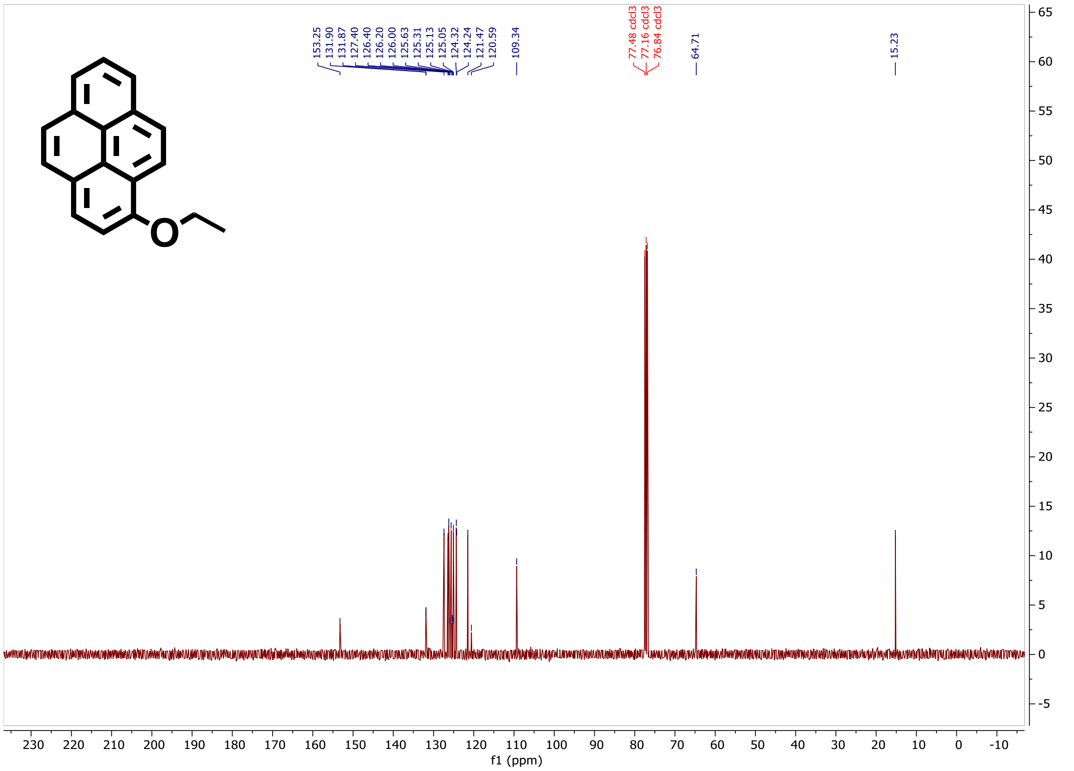

Figure S10. ^{13}C-NMR spectrum of **PyrOEt**.

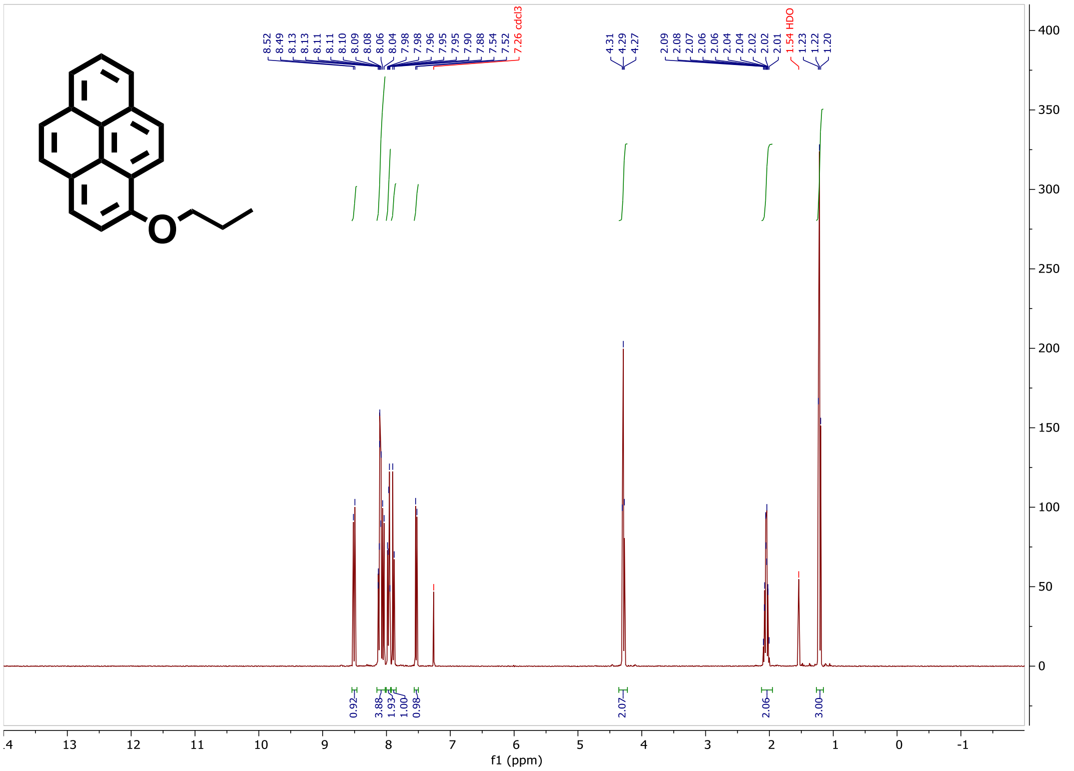

Figure S11. ^{1}H-NMR spectrum of **PyrOPr**.

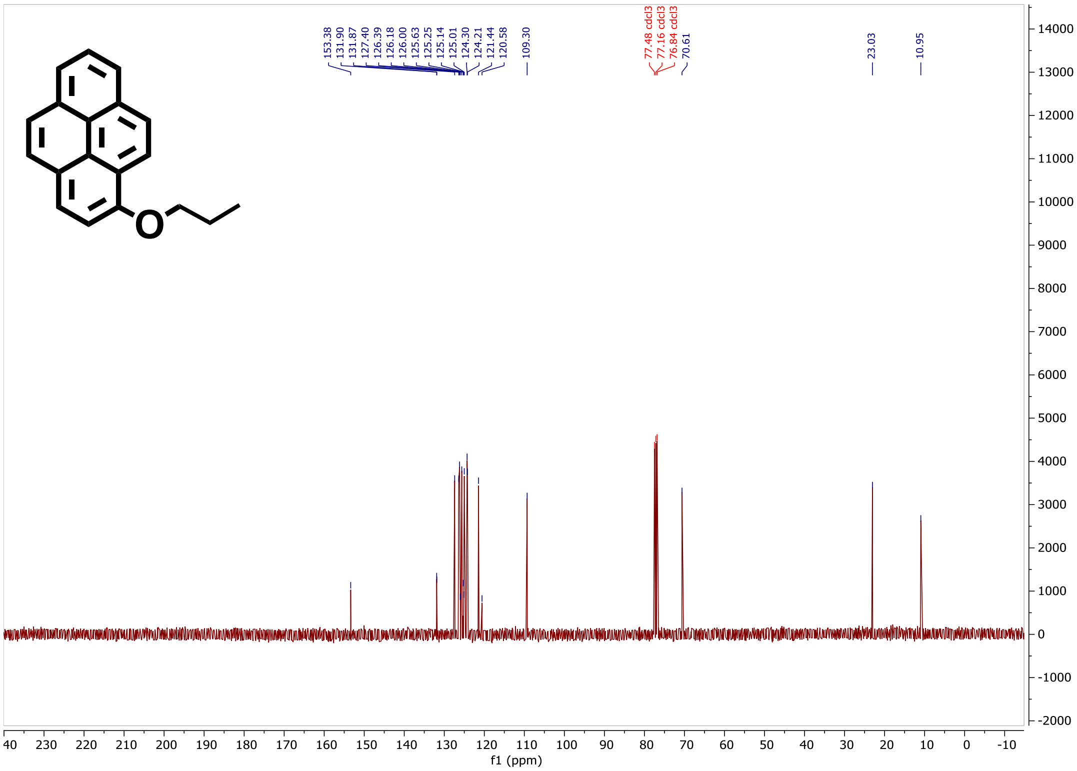

Figure S12. ^{13}C-NMR spectrum of **PyrOPr**.

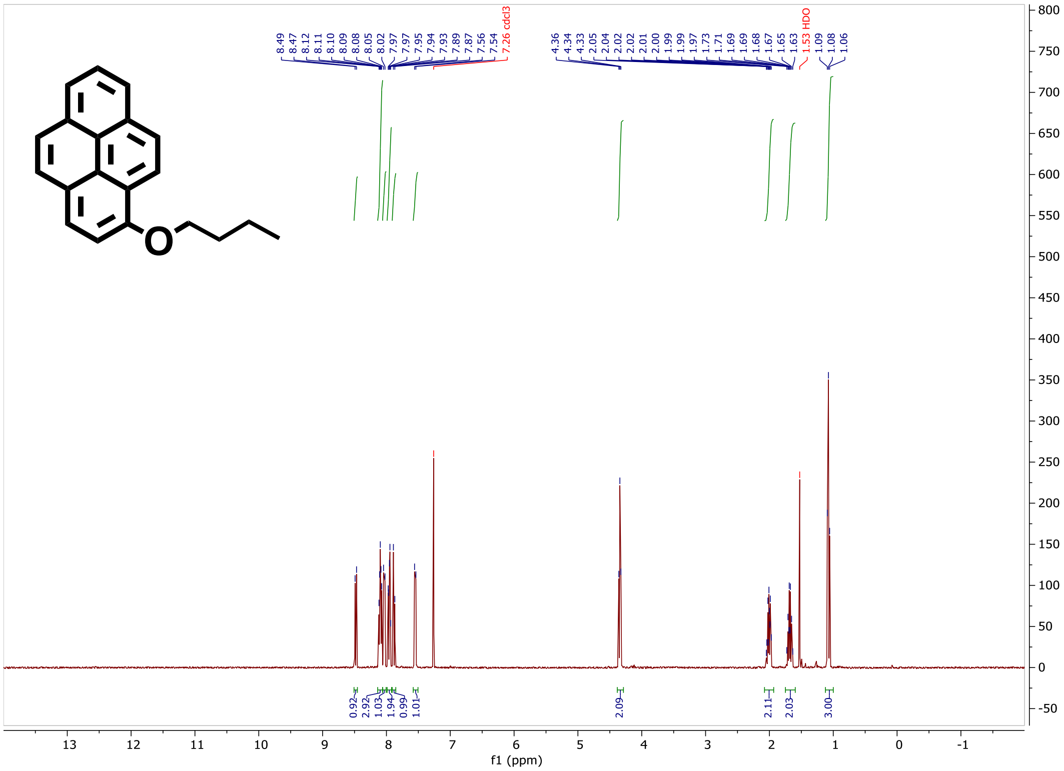

Figure S13. ^{1}H-NMR spectrum of **PyrOBu**.

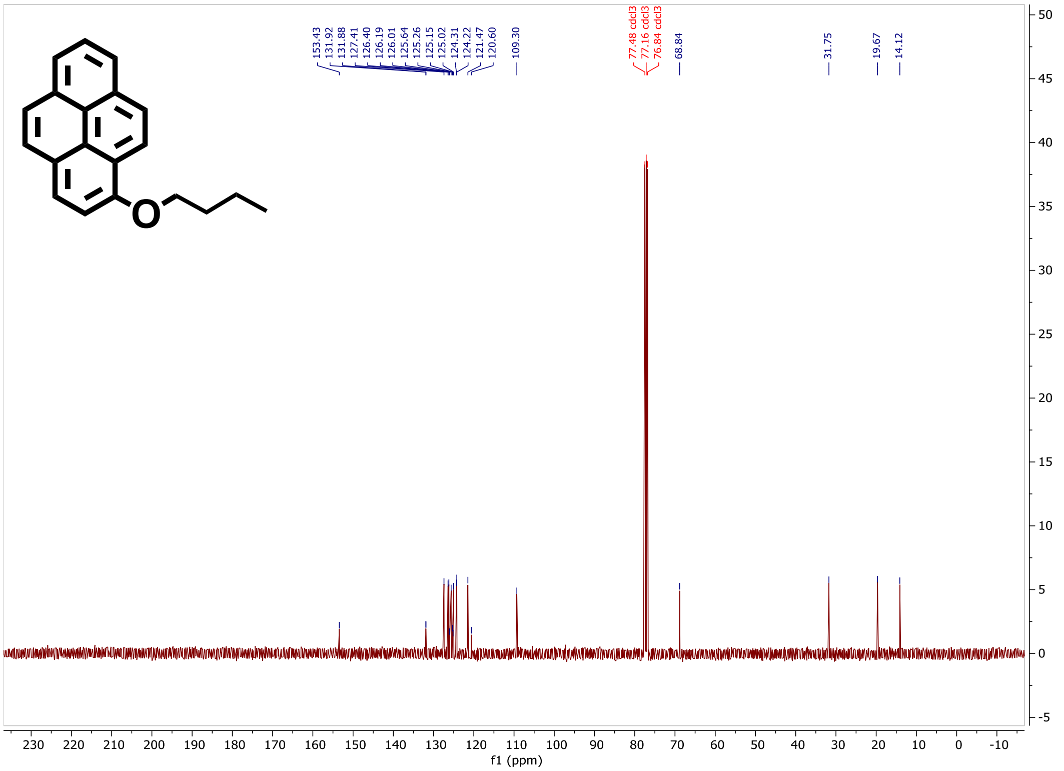

Figure S14. ^{13}C-NMR spectrum of **PyrOBu**.

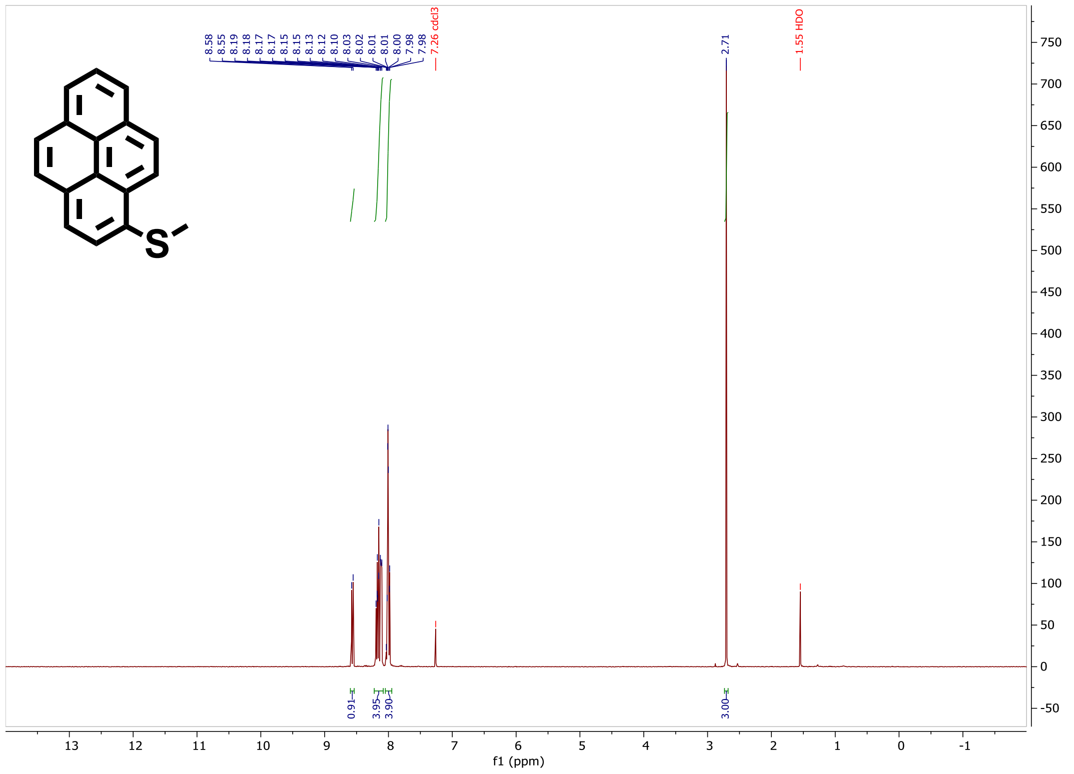

Figure S15. ^{1}H-NMR spectrum of **PyrSMe**.

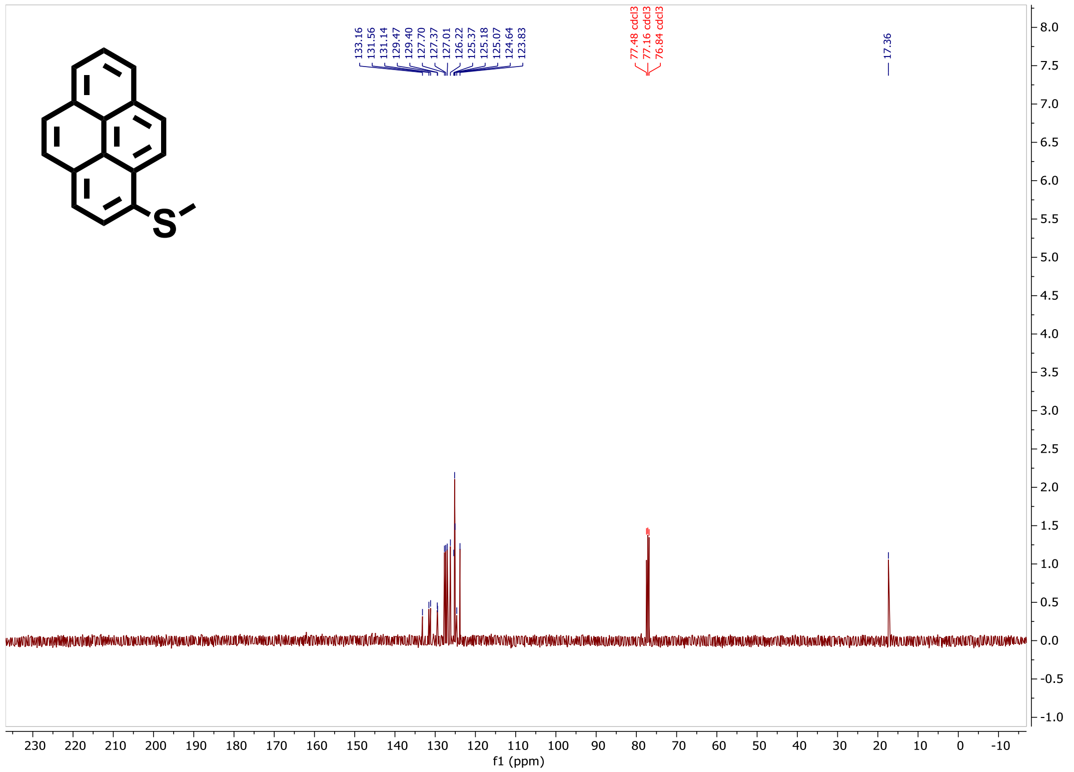

Figure S16. ^{13}C-NMR spectrum of **PyrSMe**.

References

[1] I. Horcas, R. Fernández, J. M. Gómez-Rodríguez, J. Colchero, J. Gómez-Herrero, A. M. Baro, *Rev. Sci. Instrum.* **2007**, *78*, 013705.

[2] J. A. Berrocal, G. H. Heideman, B. F. M. De Waal, M. Enache, R. W. A. Havenith, E. W. Meijer, B. L. Feringa, *J. Am. Chem. Soc.* **2020**, *142*, 4070–4078.

[3] J. A. Berrocal, G. H. Heideman, B. F. M. de Waal, E. W. Meijer, B. L. Feringa, *ACS Nano* **2020**, 10.1021/acsnano.0c06274.

[4] M. Lukeman, M.-D. Burns, P. Wan, *Can. J. Chem.* **2011**, *89*, 433–440.

[5] T. Wunder, J. Marr, S. Kremer, O. Sterner, H. Anke, *Arch. Microbiol.* **1997**, 310–316.

[6] U. Sack, T. M. Heinze, J. Deck, C. E. Cerniglia, M. C. Cazau, W. Fritsche, *Appl. Environ. Microbiol.* **1997**, *63*, 2906–2909.

[7] C. Tintel, F. J. Rietmeyer, J. Cornelisse, *Recl. Trav. Chim. Pays-Bas* **1983**, *102*, 224–228.

[8] E. Profft, R. Biela, *Chem. Ber.* **1961**, *94*, 2374–2382.

[9] J. V. Passarelli, D. J. Fairfield, N. A. Sather, M. P. Hendricks, H. Sai, C. L. Stern, S. I. Stupp, *J. Am. Chem. Soc.* **2018**, *140*, 7313–7323.

[10] R. S. Kathayat, N. S. Finney, *J. Am. Chem. Soc.* **2013**, *135*, 12612–12614.
